# A Comparative Cost Analysis of Antibiotic Treatment for Community Acquired Pneumonia (CAP) in Adult Inpatients at Piggs Peak Government Hospital in Swaziland

**DOI:** 10.3389/fpubh.2018.00303

**Published:** 2018-11-20

**Authors:** Sifundo P. Zwane, Shelley-Ann M. McGee, Fatima Suleman

**Affiliations:** Discipline of Pharmaceutical Sciences, School of Health Sciences, University of KwaZulu-Natal, Durban, South Africa

**Keywords:** CAP, pneumonia, cost, antibiotics, treatment

## Abstract

**Background:** Of the different types of pneumonia, community acquired pneumonia (CAP), has been identified as the leading cause of infectious morbidity and mortality in the western and developing countries. To eradicate the bacterial cause of CAP, medical doctors) often tend to prescribe a differing cocktail of medicine which may be costly for the health care system.

**Aim:** To analyze the cost of oral and/or intravenous antibiotic medicine use in different treatment approaches for treating CAP in adult inpatients from the health care system perspective.

**Settings:** This study was undertaken at Piggs Peak Government Hospital, a 220 bed tertiary hospital located in the rural northern Hhohho region of Swaziland.

**Method:** Seventy-one (*n* = 71) medical records of adult patients, hospitalized and diagnosed with CAP at Piggs Peak Government Hospital from July 2014 to June 2015, were retrieved and entered into the database once confirmed as having met the selection criteria. Only direct antibiotic medicine(s) costs were considered. The total cost per treatment option was calculated by multipling the unit cost of the medicine by the administration frequency and the length of hospital stay. The Kruskal-Wallis test was used to compare the cost difference between more than two treatment options.

**Results:** Medical doctors at Piggs Peak Government Hosptial use a range of antibiotics to treat community acquire pneumonia. Furthermore, doctors prefer using dual antibiotics combination as first line treatment of CAP in adult inpatients. The cost of treating community acquire pneumonia at the hospital ranged from ZAR 70.98 to ZAR 467.60 per adult inpatient admitted into care. A statistically significant difference in the cost of the different treatment approaches used for treating CAP was noted.

**Conclusion:** This cost-exploratory study has highlighted a significant difference in the monetary cost of the differing approaches used for treating CAP at the hospital. It is evident therefore that the use of different treatment approaches in treating CAP significantly influences the cost of CAP treatment. There is therefore need for cost minimization measure to be put in place at the facility.

## Introduction

Community-acquired pneumonia (CAP) is the leading cause of infectious morbidity and mortality in the western and developing countries, with the African continent carrying a substantial burden of CAP. Around 30% of the estimated 430 million LRTIs episodes reported in Africa each year are CAP (1). It is one of the most serious infectious diseases, accounting for a considerable number of hospital admissions and increased rates of serious complications.

Although an important cause of mortality and morbidity worldwide, emerging data is available on specific incidences for etiologies of acute respiratory such as CAP in children and adults in the African continent (2, 3). The 2015 Global burden of disease (GBD) study reported over 290 million cases of LRTIs worldwide, a 6.8% increase from the 2005 LRTIs incidence (4). This according to Corrêa et al. (5) accounts for 4.9% of all deaths in the world (5). Cupurdija (6) further estimated that 4–6 million of CAP cases occur in the United States annually, of which approximately 20–25 % required hospitalization (6). A study by Cajetan and Chukwuka (7) in Nigeria that reviewed 160 inpatient cases of CAP showed an 11.9% hospital mortality rate whereas a similar study in Ethiopia (1) showed 11% comparable mortality among admitted patients with CAP.

Most cases a diagnosis of CAP is made on clinical grounds and patients are often initiated on empirical antibiotic treatment before the results of laboratory tests are seen (4) or worst, if they are not done at all. Medical doctors in such instances tend to prescribe a differing cocktail of medicine in order to eradicate pneumonia (5). Many factors may contribute to the rationale behind these differing approaches. These differing treatment options though may not improve outcome of patients and may hence impact negatively on the health care cost (6).

In low income hospital settings like Swaziland where overuse and/or inappropriate use of medicines (including antibiotics), and where empirical treatment is widely practiced, this may precipitate in both patients and the health care system spending excessively on pharmaceuticals and wasting financial resource (7).

For the 2013/14 financial year, Central Medical Stores (CMS) data reflects that the government of Swaziland spent over 1 million South African Rands (equivalent to $108500) to procure essential antibiotics alone. According to B Mhlanga (Personal Communication, October 2015) of this, approximately 39% (ZAR 387 000) was calculated to have been issued for use at Piggs Government Hospital, a 220 bed region hospital located in the northern Hhhohho region of Swaziland.

To curb this inappropriate or over usage of antibiotics or drugs in general the World Health Organization (WHO) from time to time publishes a core list of minimum medicine needed for a basic health-care system (8). Listing the most efficacious, safe and cost–effective medicines for priority conditions like CAP. In the treatment of mild to moderate CAP, WHO recommends the use of amoxicillin, amoxicillin + clavulanic acid, ampicillin, or benzylpenicillin as first choice treatment. Cephalosporines, Cefotaxime and ceftriaxone, together with clarithromycin, and/or gentamicin is recommended for treatment of severe or complicated CAP in adults (9).

A study that analyses the cost of the use of antimicrobial medicine for CAP has not been done in Swaziland. As a result, it is hence difficult to determine whether the different treatment strategies employed in the treatment of CAP improve patient outcomes or they are an unnecessary burden on the country's healthcare system. The lack of such economic analyses makes it difficult to make improvements in CAP treatment strategies in the country.

This study was hence designed to determine and compare the cost associated with antimicrobial medicine used in treating CAP in adult inpatients at Piggs Peak Government Hospital. The cost comparison shall guide decision makers, medical practitioners, pharmacists and help to improve the national guidelines for the treatment of CAP in Swaziland. It shall also guide medicines budgeting both at hospital and national government level.

With no such study previously done in the country, this research hopes to form basis for later cost comparison studies in the Kingdom. It is further hoped that such study will be replicated in other hospitals in Swaziland, for different diseases and across different age groups in the Southern African Developing Countries.

## Methods

### Study design

This study was undertaken at Piggs Peak government hospital a health care facility located in the rural northern Hhohho region of Swaziland. Piggs Peak government hospital is a tertiary government referral hospital with a total bed capacity of 220. This was a retrospective study that assessed the treatment of CAP in adult male and female patients between the age of 18 and 65 years who were admitted at the hospital between July 2014 and June 2015. Retrospective patient information that was contained in the admission sheet, bed head, continuation form, nurses note, doctors' notes, treatment sheet and discharge summary of a complete patient file was retrieved and captured using a questionnaire.

### Study sample

Sample size was based on the number of adult patients admitted and diagnosed with CAP over a specific period. Medical records of adult patients hospitalized in the male and female wards were retrieved and all patients diagnosed with CAP were selected and entered into the database once confirmed to having met the selection criteria. Seventy-one suitable patient records were identified and sampled from this site.

After data collection patients were classified into treatment groups based on initial antimicrobial regimen prescribed and administered. Only antimicrobials administered within the first 36 h after hospital arrival was considered in the classification of patients into the treatment options. Costs of any subsequent treatment(s) were included based on the initial treatment classification.

### Inclusion and exclusion criteria

Eligible patients were adults 18 years or older admitted into care with a diagnosis of CAP between 1 July 2014 and 31 June 2015. Patients with the Human immunodeficiency Virus (HIV), pregnant or nursing woman, children, patients with active tuberculosis and patients with chronic kidney failure were considered ineligible and excluded from the study sample.

Analysis for CAP was limited by excluding patients who were in the hospital 14 days prior to admission for CAP. Only the first of patient's multiple hospitalisations for CAP was included for analysis. Cases of death or discharge within 24 h after admission were also excluded. Confidentiality was maintained throughout the study.

### Data collection

Data was collected over a period of three (3) months. A data collections tool was developed by the researcher to collect information on patient demographics, diagnosis, antibiotics prescribed, treatment duration, date of admission and discharge.

Continuous, categorical and nominal types of data were collected for the different variables that were examined. The hospital number instead of patient name was used for purposes of confidentiality. The date of admission and discharge were used to calculate length of hospital stay.

### Cost calculations

The study only considered antibiotic medicine costs used in the treatment of CAP. The 2014/2015 fiscal year central medical stores tender medicine cost prices were used when calculating the relevant cost of antibiotic treatment for the specific treatment duration. The quantification of costs considered for the study were medical costs associated with antibiotics used.

### Statistical analysis

For each of the study objectives data was analyzed and presented as shown in the Results section of this paper. For statistical analysis, the 2015 version of the Statistical Package for Social Science (SPSS) was used.

The total cost was calculated using information extracted from the patient's medical file. The Kruskal-Wallis test was used to compare the cost difference between more than two treatment options. Results of the different analysis and comparisons were analyzed and are presented in the Results section.

### Ethical considerations

The protocol of this study was reviewed and given full ethics approval by the Biomedical Research Ethics Committee (BREC), an ethics committee registered with the South African National Health Research Ethics Council (REC-290408-009) and in country (Swaziland) by the Swaziland Research and Ethics Council (SEC). To ensure confidentiality of information source(s), patient hospital numbers rather than names were used for patient identification.

## Results

### Patient demographics

A total of 71 (*n* = 71) patient records were identified and reviewed in this study. Forty-four (44%) percent of patients admitted with a diagnosis of CAP were male, and most of the patients were between 21 and 50 years. SPSS analysis shows the average age for this study sample to be 43 years.

Table 1 below shows that on average a person admitted with CAP will spend approximately 8 days at the hospital admitted. Furthermore, whilst hospitalized, patients are put on intravenous antibiotics for an average of 4 days.

**Table 1:** Study patient demographics.

		**Patient age**		**Age (years)**	**Days on treatment**	**Days hospitalized**
		**Female**	**Male**			
*N*	Valid	40	31	71	71	71
Mean		36	50	43	4.01	8.10
Median		33	51	43	3.50	7.00

### Antibiotic treatment options for CAP

Table 2 illustrated the various treatment options used to treat CAP by medical doctors at Piggs Peak Government Hospital. Fifteen treatment options were identified. The treatment options identified show that practitioners at PPGH use either a single or a combination of antibiotic when treating CAP. Furthermore, medical doctors at the hospital use either single or double medicine combinations in treating CAP at the hospital.

**Table 2:** Antibiotic treatment for CAP.

**Medicine treatment option**	**No. Patient(s) *N* = 71**
Benzylpenicillin + Gentamycin	24
Ceftriaxone + Genatmycin	11
Amoxicillin + Gentamycin	10
Ceftriaxone only	7
Benzylpenicillin only	6
Amoxicillin only	4
Amoxicillin + Gentamycin + Ceftriaxone	1
Benzylpenicillin + Gentamycin+ Ciprofloxacin	1
Benzylpenicillin + Amoxicillin + Gentamycin	1
Ciprofloxacin only	1
Ceftriaxone + Chloraphenicol + Gentamycin	1
ceftriaxone + Chloraphenicol	1
Benzylpenicillin + Chloramphenicol	1
Benzylpenicillin + Ceftriaxone	1
Amoxi-Clavulanic Acid + Gentamycin + Benzylpenicillin	1

Amoxicillin, ceftriaxone and benzyl penicillin are amongst the widely used antibiotics in the treatment of CAP. These it has been identified are used either alone or in combination with another antibiotic medicine.

The results show that the most preferred antibiotic combination for CAP treatment at Piggs Peak hospital is a combination of benzylpenicillin and gentamycin. Of the 71 identified case of CAP between July 2014 and June 2015, a majority (33.8%) of patients were treated with benzylpenicillin and gentamycin, 14.1% and 15.5% were each treated with a combination of either amoxicillin and gentamycin and ceftriaxone and gentamycin, respectively.

A small fraction (8.5%) of patients was treated with benzylpenicillin, which is the standard recommended treatment specified in the national standard treatment guidelines for treatment of CAP. Other single medicine regimes that were identified included amoxicillin (5.6), ciprofloxacin (1.4%) or ceftriaxone (9.9%).

### Cost of treatment options

The Shapiro-Wilk test of normality in distribution of cost of treatment in this population sample was not normal. The Shapiro-Wilk test for normal data gave a *p* > 0.001, hence the assumption of normality was rejected. Therefore, non parametric statistical tests were used.

The unit cost of medicines was obtained from medicine records kept at the Central Medical Stores. The total cost of each antibiotic medicine treatment administered was calculated using the unit dose cost multiplied by the dosing frequency and the number of days the patient was hospitalized and put on antibiotic treatment.

The median cost of treating CAP at the hospital was found to be ZAR 113.58 and the mean cost was calculated at ZAR 145.06. It is further established that it is more costly to use multiple antibiotic medicine therapy than single antibiotic therapy.

### Treatment option cost analysis

The Dunn's test on cost of medicine treatment by age group showed that there is no significant difference in the cost of treatment by age group (*p* = 0.7). The two-sample Wilcoxon rank-sum (Mann-Whitney) test showed that there is no difference in the cost of treatment by gender (*p* = 0.9).

The Kruskal-Wallis test was used to compare the differing cost of CAP treatment options. A *p*-value (*p* < 0.001) from this test suggests that the cost differs by category of treatment. To determine where this difference in treatment cost lay, the Dunn's pairwise comparison test was used.

Table 3 shows where the differences in treatment option cost lies. For example Table 3 shows that cost of treatment with Amoxicillin plus Gentamycin differs from that of treatment option benzylpenicillin + plus gentamycin. Furthermore, treatment with benzylpenicillin only and ceftriaxone + gentamycin options differs from treatment with benzylpenicillin + gentamycin and benzylpenicillin only option.

**Table 3:** Cost comparison analysis.

**Treatment cost**	***N***	**p50**	**p25**	**p75**
**Amoxicillin plus gentamycin**				
Actual cost	10	241.41	171.53	345
Std cost	10	79.8	79.8	79.8
Diff	10	161.61	91.73	265.2
**Benzylpenicillin plus genatmycin**				
Actual cost	24	48.88	40.7	87.4
Std cost	24	79.8	79.8	79.8
Diff	24	−30.92	−39.1	7.6
**Ceftriaxone plus genatmycin**				
Actual cost	11	200.45	172.5	272.92
Std cost	11	79.8	79.8	79.8
Diff	11	120.65	92.7	193.12
**Benzylpenicillin only**				
Actual cost	6	35.91	27.93	39.9
Std cost	6	79.8	79.8	79.8
Diff	6	−43.89	−51.87	-39.9
**Amoxicillin only**				
Actual cost	4	155.25	115	272.94
Std cost	4	79.8	79.8	79.8
Diff	4	75.45	35.2	193.14
**Ceftriaxone only**				
Actual cost	7	100.2	100.2	267.2
Std cost	7	79.8	79.8	79.8
Diff	7	20.4	20.4	187.4
**Other**				
Actual cost	9	140.9	95.47	206.05
Std cost	9	79.8	79.8	79.8
Diff	9	61.1	15.67	126.25

*Actual cost, cost of the treatment option used; Std cost, cost of standard treatment (benzylpenicillin only); Difference, difference between actual and standard antibiotic medicine cost*.

The Swaziland Standard Treatment Guidelines (STG, 2015) recommends the use of benzyl penicillin 5 mu four times a day for 5 days for treatment of hospitalized CAP cases. This in monetary value translates to ZAR 79.80. This cost was calculated based on unit antibiotic medicine cost, the standard recommended dosing frequency and duration of treatment duration.

The difference between actual and standard antibiotic medicine cost in USD was calculated at three different percentiles and is shown in Table 3 herein.

The difference between actual and standard was significantly different from 0 when using Wilcoxon signed-rank test for: treatment option with amoxicillin + gentamycin (Prob > |z| = 0.0050) and treatment option with ceftriaxone + gentamycin (Prob > |z| = 0.0033). These had actual cost significantly greater than standard treatment.

The Kruskal-Wallis test used to compare more than two treatment options showed a *p* < 0.001 suggesting that cost differed by treatment option. To determine where this difference in cost lay, the Dunn's pair-wise comparison test was used. Table 4 shows this pair-wise comparison and where the differences in cost lay.

**Table 4:** Dunn's Pair-wise Comparison of cost of medicine treatment.

	**Amoxicillin + Gentamycin**	**Benzylpenicillin + Genatmycin**	**Ceftriaxone + Genatmycin**	**Benzylpenicillin**	**Amoxicillin**	**Ceftriaxone**
Benzylpenicillin+Gentamycin	4.1046 **0.0004**	
Ceftriaxone+Genatmycin	0.1250 1.0000	−4.0930 **0.0004**	
Benzylpenicillin only	4.1824 **0.0003**	1.3471 0.85587	4.1479 0.0004	
Amoxicillin only	0.5959 0.9989	−2.2078 0.2504	0.5102 0.9995	−2.799 0.0523	
Ceftriaxone only	1.3248 0.8701	−2.0767 0.3304	1.2372 0.9093	−2.708 0.0686	0.4791 0.9997	
other	1.6184 0.6798	−2.0500 0.3482	1.5329 0.7430	−2.6870 0.0730	0.6507 0.9981	0.1801 1.0000

*P-values of significance are highlghted in bold*.

Table 4 shows that (i) Treatment with amoxicillin+gentamycin differed significantly from treatment with benzylpenicillin+gentamycin (*p* = 0.0004) and benzylpenicillin only (*p* = 0.0003). (ii)Treatment ceftriaxone+gentamycin differed from treatment benzylpenicillin+gentamycin (*p* = 0.0004) and benzylpenicillin only (*p* = 0.0004).

## Discussion

This study considered only level 1 costs i.e., price of antibiotic medicines used in treating CAP at a tertiary hospital.

The study has identified sixteen different antibiotic cocktails used by medical doctors when treating CAP in adult inpatients at Piggs Peak Government Hospital, the rationale of which still needs to be explored.

Amongst the antibiotic medicines recommended in the treatment of CAP, the WHO Essential Medicines List (9) of 2017, states amoxicillin, ceftriaxone, gentamicin, benzylpenicillin and amoxicillin-clavulanic as recommended molecules. It is worth noting that of the identified treatment cocktails for CAP at PPGH 99% of the cases were treated using these WHO recommended medicine either alone or in combination.

Of the identified antibiotic treatment options in this study, 27% consisted of a single antibiotic and 73% was either a combination of 2 or three antibiotics. In contrast to this, Sow in his study of comparing clinical features and outcome in Africa (Republic of Guinea) and Europe (France) highlighted a large number (90%) of cases in Guinea (Africa) (10) where CAP was being successfully treated using a single antibiotic- penicillin. This may seem to suggest that doctors at the hospital believe use of multiple antibiotics is superior over a single antibiotic option.

In the combined medicine therapy treatment options identified at facility the majority, 33% of the CAP cases were treated with a combination of injectable benzylpenicillin and gentamycin, and 15.5% were treated with a combination of ceftriaxone plus gentamycin. Both these antibiotic medicines are recommended in the latest WHO Essential Medicines List (9) for the treatment of CAP. From the analysis it is not clear whether the choice of medicine is linked to age, gender or severity of disease.

The cost of treating CAP using amoxicillin plus gentamycin differed from treating using benzylpenicillin plus gentamycin or the benzylpenicillin only option. Furthermore, the cost of treating CAP with ceftriaxone plus gentamycin differed from benzylpenicillin plus Gentamycin and the benzylpenicillin only option.

When comparing treatment cost by gender or age, it was found that there was no difference in the cost of treatment by age (p-0.7) or by gender (*p* = 0.9).

## Conclusion

This cost comparison analysis has shown that age or gender did not influence the cost of antibiotic medicine treatment, but the choice of antibiotic(s) used had an influence on the treatment cost. The study has highlighted a significant difference in the monetary cost of the differing approaches used for treating CAP at PPGH.

Treatment with (i) dual therapy -amoxicillin plus gentamycin and (ii) ceftriaxone plus gentamycin cost significantly greater when compared with the recommended standard treatment of benzylpenicillin. No significant difference in cost between the standard treatment (benzylpenicillin) and (i). benzylpenicillin plus gentamycin, (ii) amoxicillin only, and 3. ceftriaxone only treatment options was noted.

Treatment as per empirical treatment recommended in the national CAP treatment guidelines therefore cost less than the identified antibiotic used by doctors at the hospital. The rationale behind the differing antibiotic medicine choices when treating CAP needs to be explored and cost minimization measures put in place in order to contain medicine costs at the facility.

## Author contributions

All authors listed have made a substantial, direct and intellectual contribution to the work, and approved it for publication.

### Conflict of interest statement

The authors declare that the research was conducted in the absence of any commercial or financial relationships that could be construed as a potential conflict of interest.

## References

[1] Aderaye G Community acquired pneumonia in adults in Addis Abeba: etiologic agents, clinical and radiographic presentation. Ethiop Med J. (1994) 199432:115–23. 8033877

[2] Feikin DR Njenga MK Bigogo G Aura B Aol G Audi A. Etiology and incidence of viral and bacterial acute respiratory illness among older children and adults in rural western Kenya, 2007-2010. PLoS ONE (2012) 7:E43656. 10.1371/journal.pone.0043656 22937071 PMC3427162

[3] Nyamande K An approach to community-acquired pneumonia in adults. Continuing Med Educ. (2013) 31:339–41. Available online at: http://www.cmej.org.za/index.php/cmej/article/view/2859

[4] Vos T Arora M Barber RM Bhutta ZA Brown A Carter A Global, regional, and national incidence, prevalence, and years lived with disability for 310 diseases and injuries, 1990-2015: a systematic analysis for the global burdan of disease study 2015. Lancet (2016) 388:1545–602. 10.1016/S0140-6736(16)31678-6 27733282 PMC5055577

[5] Correal R de Sao Josel BP Maltall DC Passos VMA França EB Teixeira RA Burden of disease by lower respiratory tract infections in Brazil, 1990 to 2015:estimates of the Global Burden of Disease 2015 study Carga de doenca por infeccoes do trato respiratorio inferior no Brasil, 1990 a 2015:estimative do estudo Global Burden of Disease 2015. MAIO (2017) 20:171–81. 10.1590/1980-5497201700050014 28658381

[6] Cupurdija V Lazic Z Petrovic M Mojsilovic S Cekerevac I Rancic N. Community acquire pneumonia: economics of inpatient medical care vis-a-vis clinical severity. J Bras Pneumol. (2015) 41:48–57. 10.1590/S1806-37132015000100007 25750674 PMC4350825

[7] Cajetan CO Chukwuka JC Admission profile and management of community acquired pneumonia in Nigeria-5 year experience in a tertiary hospital. Respir Med. (2011) 105:298–302. 10.1016/j.rmed.2010.11.003 21112756

[8] Management Sciences for Health Managing for rational medicine use. Manag Sci Health (2012). 27.2–27.11.

[9] World Health Organization WHO Model of Essential Medicines. 20^th^ list. 2017. Available online at: http://www.who.int/medicines/publications/essentialmedicines/en/

[10] Sow O Frechet M Diallo AA Soumah S Conde MK Diot P. Community acquired pneumonia in adults: a study comparing clinical features and outcome in Africa (Republic of Guinea) and Europe (France). Thorax (1996) 51:385–58. 8733490 10.1136/thx.51.4.385 PMC1090673

